# Urban green space and happiness in developed countries

**DOI:** 10.1140/epjds/s13688-021-00278-7

**Published:** 2021-05-30

**Authors:** Oh-Hyun Kwon, Inho Hong, Jeasurk Yang, Donghee Y. Wohn, Woo-Sung Jung, Meeyoung Cha

**Affiliations:** 1 Department of Physics, Pohang University of Science and Technology, Pohang, 37673 Republic of Korea; 2 Center for Humans and Machines, Max Planck Institute for Human Development, Berlin, 14195 Germany; 3 Department of Geography, National University of Singapore, Singapore, 119260 Singapore; 4 Department of Informatics, New Jersey Institute of Technology, Newark, NJ 07103 USA; 5 Department of Industrial and Management Engineering, Pohang University of Science and Technology, Pohang, 37673 Republic of Korea; 6 Asia Pacific Center for Theoretical Physics, Pohang, 37673 Republic of Korea; 7 Data Science Group, Institute for Basic Science, Daejeon, 34126 Republic of Korea; 8 School of Computing, Korea Advanced Institute of Science and Technology, Daejeon, 34141 Republic of Korea

**Keywords:** Urban green space, Happiness, Satellite imagery, GIS

## Abstract

Urban green space is thought to contribute to citizen happiness by promoting physical and mental health. Nevertheless, how urban green space and happiness are related across many countries with different socioeconomic conditions has not been explored. By measuring the urban green space score (UGS) from high-resolution satellite imagery of 90 global cities covering 179,168 km^2^ and 230 million people in 60 developed countries, we find that the amount of urban green space and GDP are correlated with a nation’s happiness level. More specifically, urban green space and GDP are each individually associated with happiness. Yet, only urban green space is related to happiness in the 30 wealthiest countries, whereas GDP alone can explain happiness in the subsequent 30 countries in terms of wealth. We further show that the relationship between urban green space and happiness is mediated by social support and that GDP moderates this relationship. These findings corroborate the importance of maintaining urban green space as a place for social cohesion to support people’s happiness.

**Supplementary Information:**

The online version contains supplementary material available at 10.1140/epjds/s13688-021-00278-7.

## Introduction

The advantages of urban green space for public health and urban planning have been of great interest in recent years. Green spaces such as parks, gardens, street trees, riversides, and even private backyards facilitate physical activity, social events, mental relaxation, and relief from stress and heat, leading to direct and indirect benefits for mental and physical health and well-being [1–3]. Thus, worldwide policy changes and efforts have been made to provide more urban green space to create sustainable and comfortable living environments [4].

Urban green space and happiness are known to have an implicit positive correlation. Although this association is still unclear, there are at least three main reasons that relate green space with health, well-being, and happiness [5]: an innate human emotional affiliation with nature [6], reduced environmental “bads” [7, 8], and facilitating behaviors beneficial to physical and mental health. Notably, five pathways through which greenery might have beneficial effects have been reported: relieving stress, stimulating physical activity, facilitating social interactions, generating aesthetic enjoyment, and facilitating a sense of shelter from and adjustment to environmental stressors [3, 9, 10]. Studies have suggested that the same pathways exist in numerous countries [11]. While various socioeconomic factors are related to happiness [12], social interaction facilitation has been confirmed with strong evidence. Studies [13, 14] have shown that open green space promotes social cohesion by providing places for social contact; people can naturally encounter neighbors in local green spaces while walking dogs, gardening, and having outdoor parties, which enhances community engagement. Moreover, larger green areas such as parks can hold public events and activities, enabling social mixing between communities.

The amount of urban green space can be captured mainly by three kinds of measurements: qualitative ratings of observers [9, 15], national land-use and land-cover databases [5, 16, 17], and geographic information system (GIS) techniques. Among these measurements, GIS techniques are the most recently developed method. One example is utilizing the normalized difference vegetation index (NDVI), a vegetation index computed from Landsat series satellite images (30 m resolution) [10, 11, 18]. Researchers such as Tsai *et al*. [19] introduced multiple landscape metrics based on GIS and showed a strong association between green space and mental health in U.S. metropolitan areas. These studies assume that the distance from an individual’s residence to the nearest green space has associations with health data [3, 20]. The green space level was then measured as the fraction of areas with NDVI values above a certain threshold (e.g., 0.2 to 0.4 for sparse vegetation and 0.6 for highly dense vegetation) [21]. However, this method raises the question of how to set an appropriate NDVI threshold for global cities.

Despite the rich literature on the mental benefits of green space, research is limited in terms of global-scale comparative research [2]. First, the analytical settings are based on a limited number of Western countries [10]; most of these studies have been conducted in the United States [18, 19] and Europe [3, 11]. Moreover, only a few are based on multi-country settings that enable comparative analysis [22]. As a result, it is unclear whether the association between green space and mental health is robust in developing countries or only in developed countries. The main limitation arises because there is no global medical dataset providing reliable and standardized mental health surveys from different countries. Moreover, no studies have established which green space measurement is appropriate for analysis across countries. Various methods of measuring green space – questionnaires, qualitative interviews, satellite images, Google Street View images, and even smartphone technology [23] – still rely on individual-level measurements (e.g., calculating the greenery level around residential buildings) and hence are not scalable to the global level.

This paper presents a new way to analyze the effects of green space on happiness at the planetary scale, incorporate socio-economic contexts of different countries, and achieve robust results. First, we measure the amount of urban green space from high-resolution satellite images for different countries by developing a globally comparable green space metric. Our metric based on the total NDVI of built-up areas enables this comparison as it does not require an arbitrary threshold that varies for different regions. It also overcomes the limitations of official statistics based on national land-use land cover data that tend to have different criteria by countries and often include only official parks and open space. Our analysis on high-resolution (10 m) Sentinel-2 satellite images provides more accurate information on urban green space than previous studies on Landsat series images (i.e., a resolution of 30 m) [3, 10, 11, 18].

Next, this study uses selected happiness scores from the World Happiness Report [24], which provides reliable and standardized data on multiple countries’ happiness and allows comparisons among nations. As happiness is a criterion of how people feel about the course of their life, it is interconnected with various socioeconomic variables. From the perspective that economic studies distinguish between emotional well-being and life satisfaction [25], we focus on the impact of green space on life satisfaction. Specifically, we study this relationship in developed countries with the highest Human Development Index (HDI), where green environments in cities are considered more important for life satisfaction.

Using these datasets from satellite imagery, we explore the relationship between urban green space and happiness globally. Additionally, we identify conditional indirect effects by national wealth and social support by employing a moderated mediation regression model on socioeconomic indicators.

## Urban green space and happiness in countries

We examine the global relationship between urban green space and happiness in 60 developed countries ranked by the Human Development Index. Note that the term happiness represents how people feel about the course of their life [24]. Using the Sentinel-2 satellite imagery dataset, we define each country’s urban green space score (UGS) as the logarithmic total vegetation index per capita in the most populated cities (i.e., those that include at least 10% of the national population). Among the various vegetation indices available such as the NDVI, soil-adjusted vegetation index (SAVI) and two-band enhanced vegetation index (EVI2), the NDVI [26] is used based on the robustness of the results for different tested indices. The happiness score and the gross domestic product based on purchasing power parity (GDP (PPP)) per capita of each country are from the World Happiness Report [24] and the International Monetary Fund (IMF) estimation [27], respectively (see the Methods section and Additional file 1 for details).

**Figure 1:**
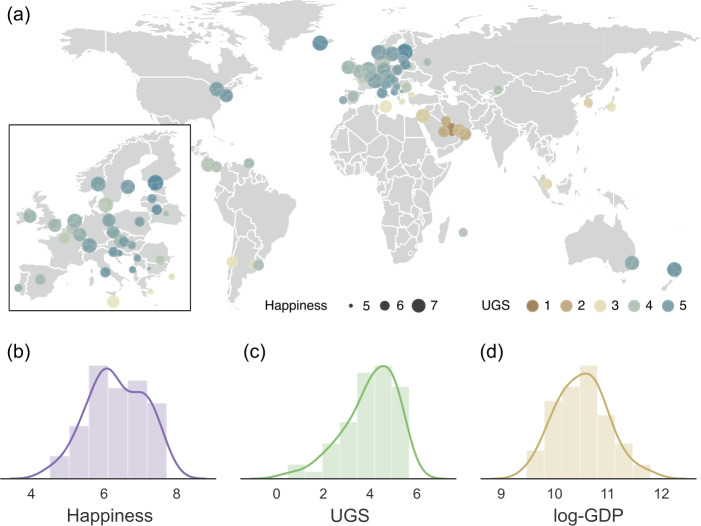
The distributions of urban green space and happiness over the world. (**a**) The map of urban green space and happiness in 60 developed countries. The size and color of circles represent the level of happiness and urban green space in a country, respectively. The markers are placed on the most populated cities of each country. (**b**)–(**d**) The histograms of (**b**) happiness, (**c**) urban green space (UGS) and (**d**) logarithmic GDP per capita (log-GDP). We use the logarithm of the total NDVI per capita as an indicator of urban green space, and the logarithm of GDP per capita as a measure of wealth

Figure 1(a) shows an overall view of urban green space and the happiness of countries worldwide. This map highlights regional differences in the green space distribution due to climate; countries near the Equator in tropical climates have relatively high UGSs, while countries located in the 20–30 latitude range have exceptionally low UGSs due to the dry climate. The UGS increases with latitude in higher-latitude regions. On the other hand, Northern and Western European and North American countries display relatively high happiness scores. Western Asian countries also show relatively high happiness with a low UGS, indicating that the relationship between happiness and green space is not trivial.

Figure 1(b)–(d) shows the distributions of happiness, UGSs, and log-GDP, and they all show unimodal distributions, which is appropriate for linear regression analyses. Note that the probability distributions of GDP per capita converge to a normal distribution after logarithmic scaling. Our comparison of several green space measures shows that the logarithmic NDVI per capita is most suitable for the following analysis in terms of its distribution and explanatory power. Therefore, we choose the logarithmic NDVI per capita as the primary green space indicator in this research (see Additional file 1). We also use the logarithmic GDP per capita (PPP) (hereinafter referred to as the log-GDP) as a measure of the wealth of the country, as noted in the Happiness Report [24].

As per-country wealth is an important indicator of citizens’ quality of life, wealth (i.e., log-GDP) should be considered when analyzing urban green space and happiness. Our regression analysis finds that the UGS, together with log-GDP, is related to happiness. We make new observations from Table 1. Although the UGS is not substantially correlated with happiness in the simple linear regression (i.e., model (2)), the multilinear model with log-GDP (i.e., model (3)) has a substantial increase in prediction ability compared to the simple regression analysis on log-GDP (i.e., model (1)). Therefore, urban green space adds explanatory power to the correlation between wealth and happiness across countries. The regression analyses with other green space-variant measures further confirm the robustness of this result, showing a substantial increase in the adjusted R-squared value when the UGS is included in the regression. Specifically, the UGS based on the logarithmic NDVI per capita shows the best regression performance. Moreover, this relationship between the UGS and happiness is robust for the control variables, including life expectancy, health expenditure, unemployment, gender inequality, and education (see Additional file 1 for the results for the different measures).

**Table 1:** The regression analysis for the UGS, happiness and log-GDP. The values denote the regression coefficients and the confidence intervals of each independent variable with its significance (i.e., ^∗∗∗^$p<0.01$; ^∗∗^$p<0.05$; ^∗^$p<0.1$). The regression model (1-3), model (4-6) and model (7-9) are examined for the data of all countries, the lower 30 countries and the top 30 countries ranked by GDP, respectively

Countries	All			Lower 30			Top 30		
Model	(1)	(2)	(3)	(4)	(5)	(6)	(7)	(8)	(9)
log-GDP	1.0120^∗∗∗^	–	1.1319^∗∗∗^	0.9034^∗∗^	–	0.8517^∗^	−0.0809	–	0.2581
	(0.6603)		(0.6234)	(1.6305)		(1.7493)	(1.3559)		(1.0314)
UGS	–	0.1165	0.2249^∗∗∗^	–	0.1497	0.0567	–	0.2785^∗∗∗^	0.2946^∗∗∗^
		(0.3545)	(0.2643)		(0.6042)	(0.6051)		(0.2313)	(0.2403)
Const	−4.2945^∗∗^	5.9007^∗∗∗^	−6.4709^∗∗∗^	−3.3428	5.1767^∗∗∗^	−3.0629	7.7712^∗∗^	5.8110^∗∗∗^	2.9312
	(6.9672)	(1.4910)	(6.8998)	(16.5490)	(2.6490)	(17.1094)	(14.8065)	(0.9455)	(11.5463)
Adjusted $R^{2}$	0.3832	0.00123	0.4786	0.1296	0.0012	0.1013	−0.0335	0.4457	0.4468
Observations	60	60	60	30	30	30	30	30	30

## Urban green space is effective in rich countries

Our results show that happiness is correlated with urban green space and the GDP of a country. However, is this green space-happiness relation uniform across all countries? Previous studies on the marginal effect of income on happiness suggest that happiness may have a nonlinear relationship with GDP, presumably showing saturation after a specific GDP – a concept known as the Easterlin paradox [28]. This paradox tells us that increases in happiness through GDP reach a saturation point, yet what factors improve happiness beyond the saturation point is unknown.

**Figure 2:**
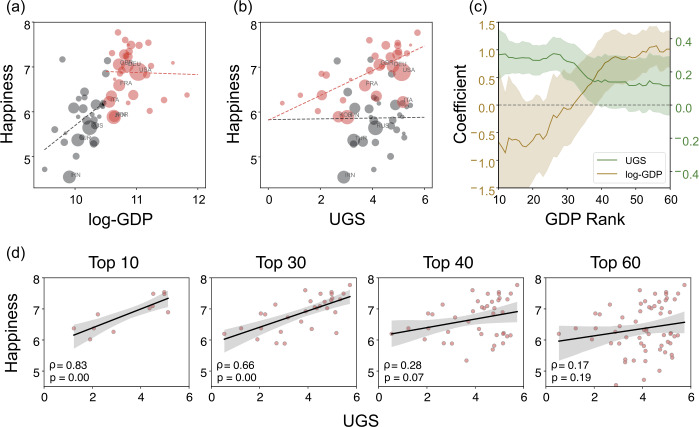
The effect of GDP on the green-happiness relation. (**a**), (**b**) The relations of (**a**) log-GDP and happiness, and (**b**) urban green space (i.e., UGS) and happiness across 60 developed countries. The top 30 and the lower 30 countries ranked by GDP are sized by the population size and colored by red and black. The dotted lines are the linear fit for each GDP group. (**c**) Changes of coefficients between urban green space and happiness for different sets of GDP rank with increasing window size from top 10 to 60. (**d**) The rank correlations between the UGS and happiness for the groups of increasing countries in the GDP rank order

To test the Easterlin paradox, we repeated the analysis over clusters of countries grouped by GDP. Figure 2(a) shows a high correlation between GDP and happiness in the 30 lower-GDP countries (i.e., $\rho = 0.40$). In contrast, the correlation is no longer evident in the 30 higher-GDP countries (i.e., $\rho = - 0.04$). These results suggest that economic prosperity (as measured by GDP) is crucial for people’s happiness but fails to explain happiness in rich countries. The GDP appears to reach a happiness-correlation threshold around the 30th wealthiest country, which corresponds to a GDP of 38,518 dollars. Previous research on the Easterlin paradox has shown that GDP per capita can increase happiness until it reaches a certain threshold but cannot further increase happiness above that threshold. We observe a similar pattern for wealth and happiness across countries. On the other hand, happiness in the 30 wealthiest countries is well explained by urban green space. As shown in Fig. 2(b), urban green space is positively correlated with happiness in the richest countries (i.e., $\rho = 0.66$, $p < 0.01$), but this correlation is not significant in the 30 lower-GDP countries (i.e., $\rho = 0.19$, $p = 0.32$). Thus, urban green space is a factor that further increases the happiness of a country after its GDP reaches a certain level.

The regressions for each of the 60 countries ranked by GDP in Table 1 confirm the individual effects of urban green space and GDP on happiness. GDP is the only substantial factor explaining happiness in the 30 lower-GDP countries (models 4-6). In contrast, for the 30 higher-GDP countries, happiness is associated only with the UGS (7-9). These findings suggest that GDP is critical for happiness until it reaches a certain GDP threshold (i.e., the Easterlin paradox), after which urban green space better explains happiness.

The correlation between the UGS and happiness also corroborates the effect of the UGS in rich countries. The correlation in Fig. 2(d) decreases as more countries in decreasing order of GDP are added. The correlation is substantial (i.e., *ρ* is approximately 0.8) among the countries excluding the top 30. Figure 2(c) summarizes the effects of urban green space and GDP, which intersect with each other around the 30th wealthiest country. For the top 30 countries, urban green space has positive coefficients, but the GDP effect is not significant. These relationships are reversed for less affluent countries.

In summary, economic support seems to be associated with happiness until the essential requirements and living standards are met. However, economic growth is not persistently linked to increasing happiness. After a certain level, urban green space appears to be related to happiness via other social factors.

## Urban green space for social cohesion

**Figure 3:**
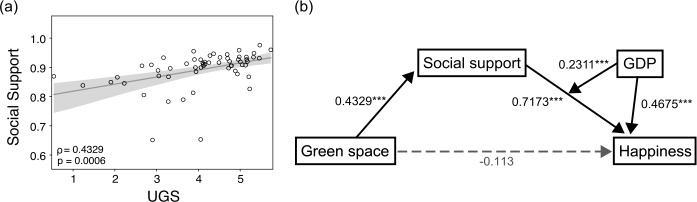
The moderated mediation model for the UGS, happiness and socioeconomic indicators. (**a**) Scatter plot of social support and the UGS across countries. (**b**) Diagram for the moderated mediation model. The boxes denote the model variables. Solid black arrows denote a statistically significant relationship between a pair of variables with the regression coefficient and the p-value (i.e., ^∗∗∗^$p<0.01$). The gray dashed arrow represents a non-significant relationship. Note that the coefficients are calculated with z-scores of the variables to compare the effect size directly

Our findings highlight urban green space as an indicator that might be correlated with social factors explaining happiness beyond achieving economic success. The question then arises: which social factors connect urban green space with happiness? To identify this connection, we first examine the correlation between the UGS and the socioeconomic variables reported in the World Happiness Report: GDP per capita, social support, life expectancy, freedom, generosity, and perceptions of corruption. Of these six variables, only “social support” has a significantly positive correlation ($\rho = 0.43$, $p < 0.01$) with the UGS, as shown in Fig. 3(a), implying that social support could mediate the relationship between urban green space and happiness. This relationship is consistent with several existing studies suggesting that urban green space is a place of social cohesion [13, 14]. Additionally, the relationship is robust for other control variables, including health expenditure, unemployment, gender inequality, and education. On the other hand, as indicated by life expectancy, physical health does not display a significant relationship with green space ($\rho = 0.19$, $p = 0.15$), contradicting common sense. The regression analysis on happiness with the UGS and six socioeconomic variables also captures the interchangeability of urban green space and social support (see Additional file 1 for details). Therefore, social support is the major component for connecting urban green space and happiness, and we construct a more elaborate model only with social support.

Here, we employ a moderated mediation model [29] to characterize the complicated relationships among urban green space, social support, GDP, and happiness. In moderated mediation models, the moderator describes a variable’s conditional effect through the interaction term, and the mediator describes a variable’s indirect effect connecting the other two variables. Accordingly, moderated mediation models determine the pathway of directed interactions between multiple variables.

First, we examine the mediation effect of urban green space and social support as independent variables. The mediation regression model shows that social support mediates the relationship between urban green space and happiness such that (1) urban green space improves social support and (2) social support is positively correlated with happiness. The mediation effect is significant (3) only when GDP is considered in the model. Consequently, our moderated mediation model combining these three effects, shown in Fig. 3(b), presents the pathway by which green space is related to happiness through social support, given that GDP moderates the effect of social support. If we describe this relationship in equations,

1 $$\begin{aligned}& H = \beta _{0} + \beta _{1} M + \beta _{2} S + \beta _{3} SM, \end{aligned}$$

2 $$\begin{aligned}& S = \beta _{4} + \beta _{5} \ln {G}, \end{aligned}$$

where *H*, *M*, *S*, and *G* represent happiness, log-GDP, social support, and the NDVI per capita, respectively, and the *β* values denote the regression models’ coefficients (see Additional file 1 for details).

Our moderated mediation model can be used to estimate the amount of urban green space that corresponds to a certain amount of increase in happiness according to

3 $$ \Delta H = \bigl( \beta _{1}'+\beta _{2}'M \bigr)\ln { \frac{G_{f}}{G_{i}}}. $$

In Equation (3), the required ratio of urban green space in a country decreases as its log-GDP increases. The required increase in urban green space per capita can be estimated for each country based on its current GDP value. For example, in the United States, an increase in the NDVI per capita of urban green space of 36.1908 corresponds to an increase in the happiness score increase of 0.0546. In contrast, 3416 USD per capita corresponds to the same increment in happiness. We used a 0.0546 happiness score as a reference value of Δ*H*, the average value between happiness ranks. Note that the NDVI per capita is interpreted as a weighted area of green space, with a unit of $m^{2}$. Similarly, Qatar needs 0.4981 NDVI per capita or 7556 dollars per capita, and South Korea needs 4.1332 NDVI per capita or 2315 dollars per capita to achieve the reference happiness score increase.

## Discussion

This paper revealed a global relationship between urban green space and happiness in over 60 countries using high-resolution satellite imagery. Urban green space shows strong correlations with happiness in developed countries (i.e., countries with higher GDPs), which suggests that urban green space is a key to understanding happiness beyond economic success. Our moderated mediation model further elucidates this relationship as social support mediates the green-happiness relationship and GDP moderates social support and happiness. This sophisticated model can estimate the differential amount of additional green space corresponding to the same increment of happiness in each country.

The current study newly defined the UGS concept, which can be used to calculate the amount of green space at any spatial scale accounting for population density. We compared several green space measures and proposed using the logarithmic NDVI per capita as a preferred measure of the UGS. This index was validated through experiments and it makes it possible to investigate green space at a global level, allowing us to perform cross-sectional research on green space. Furthermore, the method of obtaining the UGS can be utilized to investigate any spatial areas such as blue space (i.e., aquatic environments such as lakes and shores) [30, 31].

Our findings have multiple policy-level implications. First, public green space should be made accessible to urban dwellers to enhance social support. In doing so, one critical aspect is public safety. If public safety in urban parks is not guaranteed [32, 33], its positive role in social support and happiness may diminish. The meaning of public safety may change; for example, ensuring biological safety will be a priority in keeping urban parks accessible during the COVID-19 pandemic [34]. The high indoor transmission rate of the virus [35] will increase awareness and the importance of open spaces such as urban parks. While some urban parks may be closed during lockdowns, some reports suggest that viewing them from home could also help reduce stress during the pandemic [36]. Second, urban planning for public green space is needed for both developed and developing countries. While our findings confirmed a strong correlation between urban green space and happiness in developed countries, the same positive relation holds for developing countries, albeit to a smaller degree. Furthermore, it is challenging or nearly impossible to secure land for green space after built-up areas are developed in cities. Therefore, urban planning for parks and green recovery (new greening in built-up areas) should be considered in developing economies where new cities and suburban areas are rapidly expanding [37, 38].

In addition to the above, recent climate changes can create substantial volatility in sustaining urban green space. Extreme events such as wildfires, floods, droughts, and cold waves could endanger urban forests around the world [39]. On the other hand, global warming could also accelerate tree growth in cities more than in rural areas due to the urban heat island effect [40]. Ultimately, the environmental influence is bidirectional; urban green spaces affect local climates by reducing carbon dioxide levels [41] and providing a cooling effect inside the city that indirectly affects people’s well-being. Thus, more attention must be paid to predicting climate changes and discovering their impact on public places since extreme changes could hamper the benefits of urban green space.

As an exciting future direction, satellite images of higher spatiotemporal resolutions can be used to compute urban green space scores. This paper focused on the correlation across countries fixed in time, given the short span of the Sentinel-2 dataset launched in 2015. A causal analysis could be performed with satellite imagery data for a longer span. Additionally, our dataset does not cover all countries in the world. Fortunately, our observations from the 30 lower-income countries anticipate the substantial effect of GDP in other developing countries excluded in our analysis. Although we analyzed the highest-resolution public dataset of satellite imagery in this study, our method still has room for application to higher-resolution nonpublic datasets such as the household level (less than 10 m resolution) available in national-scale health datasets [42]. Since satellite imagery cannot account for green space inside buildings (such as green walls), future research could quantify the effect of these mini-scale green spaces using computer vision [43].

Finally, this study is not without limitations. Our country-scale analysis inevitably has a small size of data, while our findings are statistically significant. A dataset with different levels of coverage and data on happiness at the national level and urban green space in cities that contain at least 10% of the population may also include a potential bias due to within-country heterogeneity. Better accessibility to satellite imagery data at different scales and high-resolution global happiness data will enable a better estimation accounting for variations across different cities in a country.

## Methods

### Collecting happiness and remote sensing data

We use the happiness scores from the World Happiness Report [24] and the NDVI scores from Sentinel-2 satellite imagery as remote sensing data to identify the relationship between happiness and green space. The 2018 World Happiness Report covered 156 countries. The report provides an annual survey of how happy citizens perceive themselves to be and ranks the countries by their *happiness scores*. The score is the average of the participants’ responses on how happy they are on a scale from 0 to 10. While many socioeconomic indicators (e.g., unemployment and inequality) may affect happiness, not all of these factors are measured annually across 156 countries. Instead, the report describes happiness with six primary socioeconomic indicators: GDP per capita, social support, life expectancy, the freedom to make life choices, generosity, and perceptions of corruption. For example, the social support variable is based on binary responses (yes/no) to a Gallup World Poll question: “If you were in trouble, do you have relatives or friends you can count on to help you whenever you need them or not?”

To quantify urban green space in global cities, we use the Sentinel-2 dataset, which provides the highest spatial resolution (10 m) among the publicly available satellite imagery datasets (e.g., 30 m resolution in Landsat series) [3, 10, 11, 18]. With this high resolution, we can identify granular green space, including street vegetation and home gardens that could not be detected in other public datasets. When using satellite imagery to detect small-scale vegetation, it is critical to consider the season in which the images were obtained [3, 10, 11, 18]. We used images from summer: June to September 2018 for the Northern Hemisphere and December 2017 to February 2018 for the Southern Hemisphere. Satellite images with less than 10% cloud cover were used; when such images could not be obtained for the study period, data from 2019 were used instead.

The NDVI is a well-known remote sensing indicator of green vegetation areas in satellite images [26]. It detects vegetation as the difference between near-infrared and red light in the value range from −1 to +1. High NDVI scores include urban green spaces such as official parks, backyards, street trees, mountains, riverbanks, golf courses, and urban farmlands. There are a few well-known variants of NDVI [23], such as the SAVI [44], which is corrected for soil brightness, and the enhanced vegetation index (EVI) [45], which is corrected for atmospheric effects. All NDVI, SAVI, and EVI2 scores can be calculated from the two spectral bands of Sentinel-2, red (band 4) and near-infrared (NIR, band 8), as follows:

4 $$\begin{aligned}& NDVI = \frac{NIR - RED}{NIR + RED}, \end{aligned}$$

5 $$\begin{aligned}& SAVI = \frac{(1 + L)(NIR - RED)}{NIR + RED + L}, \end{aligned}$$

6 $$\begin{aligned}& EVI2 = 2.5\frac{NIR - RED}{NIR + 2.4 RED + 1}. \end{aligned}$$

The robustness of the results based on the three green space measures was verified using the NDVI as the primary metric.

### Measuring the amount of green space

**Figure 4:**
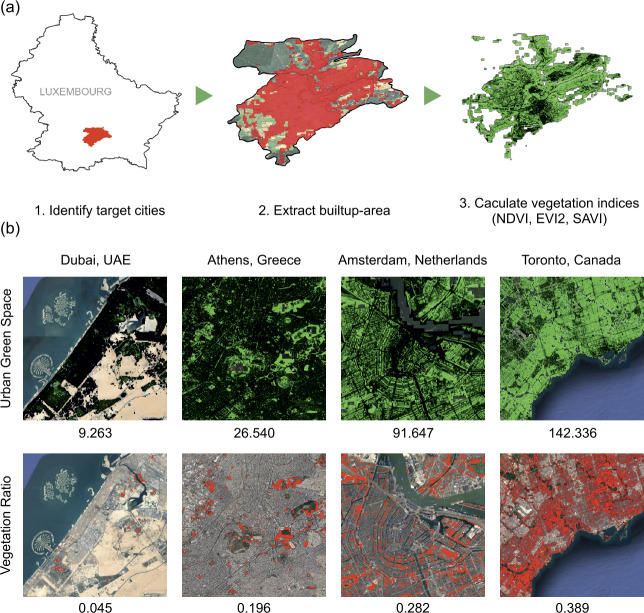
Measuring urban green spaces. (**a**) Measurement methods to compute the size of urban green space in each country. First, we find cities occupying more than 10 percent of the total population in a country. Then, we extract the built-up area of the cities with Copernicus global landcover data. Finally, we calculate vegetation indices (e.g., NDVI) within the area using Sentinel-2 satellite images. (**b**) Urban green space measured by the UGS (upper row) and the vegetation ratio (lower row) in four world cities. The red areas in the upper row indicate vegetation for the NDVI threshold of 0.4. The lower row images show the adjusted NDVI per capita (i.e., UGS) for every 10m by 10m pixel

The vegetation indices are measured in three steps, as illustrated in Fig. 4(a). The first step is to identify target cities containing at least 10% of each country’s total population and represent the country’s overall happiness. The second step is to extract only the built-up areas within the identified cities’ administrative boundaries. As the boundaries of cities are historically and culturally constructed and are often arbitrary, the cities’ size needs to be standardized; some cities include vast suburban areas (e.g., Istanbul) or natural areas (e.g., deserts in Dubai). Thus, referring to global land cover data from the EU’s Copernicus Programme [46], we focus on urban built-up areas to quantify urban green space. Finally, the vegetation indices (NDVI, EVI2, and SAVI) are calculated for the extracted urban areas.

The final step is to compute the amount of green space in each country, determined from the measured vegetation indices. Here, we define the amount of green space as the logarithm of the total NDVI of built-up areas in the target cities divided by the cities’ total population, called the UGS, as a metric for urban green space. The UGS is calculated as follows:

7 $$ UGS = \log { \biggl(\frac{\sum_{c}\sum_{b(c)}NDVI(b)}{\sum_{c}N_{c}} \biggr)}, $$

where $NDVI(b)$ is the NDVI value of pixel *b* within built-up areas $b(c)$ in city *c* and $N_{c}$ is the population of city *c*. In this calculation, we adjusted negative NDVI values to zero [23] to prevent errors caused by the accumulation of negative values in areas next to bodies of water (see Additional file 1 for the entire dataset).

## Supplementary Information

Below is the link to the electronic supplementary material.

Supporting figures and tables for the main manuscript (PDF 679 kB)

## Data Availability

Data is available on Supplementary Information.

## Abbreviations

UGS: Urban Green Space
GDP: Gross Domestic Product
GIS: Geographic Information System
NDVI: Normalized Difference Vegetation Index
SAVI: Soli-Adjusted Vegetation Index
EVI: Enhanced Vegetation Index

## References

[1] de Vries S, Verheij RA, Groenewegen PP, Spreeuwenberg P (2003). Natural environments—healthy environments? An exploratory analysis of the relationship between greenspace and health. Environ Plann A Econ Space.

[2] Gascon M, Triguero-Mas M, Martínez D, Dadvand P, Forns J, Plasència A, Nieuwenhuijsen MJ (2015). Mental health benefits of long-term exposure to residential green and blue spaces: a systematic review. Int J Environ Res Public Health.

[3] Dadvand P, Bartoll X, Basagaña X, Dalmau-Bueno A, Martinez D, Ambros A, Cirach M, Triguero-Mas M, Gascon M, Borrell C, Nieuwenhuijsen MJ (2016). Green spaces and general health: roles of mental health status, social support and physical activity. Environ Int.

[4] UN (2015) Sustainable Development Goals. https://sdgs.un.org/goals. Accessed 4 November 2020

[5] MacKerron G, Mourato S (2013). Happiness is greater in natural environments. Glob Environ Change.

[6] Falk JH, Balling JD (2010). Evolutionary influence on human landscape preference. Environ Behav.

[7] Passchier-Vermeer W, Passchier WF (2000). Noise exposure and public health. Environ Health Perspect.

[8] Welsch H (2006). Environment and happiness: valuation of air pollution using life satisfaction data. Ecol Econ.

[9] de Vries S, van Dillen SME, Groenewegen PP, Spreeuwenberg P (2013). Streetscape greenery and health: stress, social cohesion and physical activity as mediators. Soc Sci Med.

[10] Liu Y, Wang R, Grekousis G, Liu Y, Yuan Y, Li Z (2019). Neighbourhood greenness and mental wellbeing in guangzhou, China: what are the pathways?. Landsacpe Urban Plan.

[11] Dzhambov A, Hartig T, Markevych I, Tilov B, Dimitrova D (2018). Urban residential greenspace and mental health in youth: different approaches to testing multiple pathways yield different conclusions. Environ Res.

[12] Rugel EJ, Carpiano RM, Henderson SB, Brauer M (2019). Exposure to natural space, sense of community belonging, and adverse mental health outcomes across an urban region. Environ Res.

[13] Maas J, van Dillen SME, Verheij RA, Groenewegen PP (2009). Social contacts as a possible mechanism behind the relation between green space and health. Health Place.

[14] Jennings V, Bamkole O (2019). The relationship between social cohesion and urban green space: an avenue for health promotion. Int J Environ Res Public Health.

[15] Kweon BS, Sullivan WC, Wiley AR (1998). Green common spaces and the social integration of inner-city older adults green common spaces and the social integration of inner-city older adults. Environ Behav.

[16] Maas J, Verheij RA, Groenewegen PP, de Vries S, Spreeuwenberg P (2006). Green space, urbanity, and health: how strong is the relation?. J Epidemiol Community Health.

[17] Alcock I, White MP, Wheeler BW, Fleming LE, Depledge MH (2014). Longitudinal effects on mental health of moving to greener and less green urban areas. Environ Sci Technol.

[18] Beyer KMM, Kaltenbach A, Szabo A, Bogar S, Nieto FJ, Malecki KM (2014). Exposure to neighborhood green space and mental health: evidence from the survey of the health of Wisconsin. Int J Environ Res Public Health.

[19] Tsai W-L, Mchale MR, Jennings V, Marquet O, Hipp JA, Leung Y-F, Floyd MF (2018). Relationship between characteristics of urban green land cover and mental health in U.S. metropolitan areas. Int J Environ Res Public Health.

[20] Stigsdotter UK, Ekholm O, Schipperijn J, Toftager M, Kamper-Jørgensen F, Randrup TB (2010). Health promoting outdoor environments - associations between green space, and health, health-related quality of life and stress based on a Danish national representative survey. Scand J Soc Health.

[21] EOS (2019) NDVI FAQ: all you need to know about NDVI. https://eos.com/blog/ndvi-faq-all-you-need-to-know-about-ndvi/. Accessed 22 June 2020

[22] van den Berg M, van Poppel M, van Kamp I, Andrusaityte S, Balseviciene B, Cirach M, Danileviciute A, Ellis N, Hurst G, Masterson D, Smith G, Triguero-Mas M, Uzdanaviciute I, de Wit P, van Mechelen W, Gidlow C, Grazuleviciene R, Nieuwenhuijsen MJ, Kruize H, Mass J (2016). Visiting green space is associated with mental health and vitality: a cross-sectional study in four European cities. Health Place.

[23] Markevych I, Schoierer J, Hartig T, Chudnovsky A, Hystad P, Dzhambov AM, de Vries S, Triguero-Mas M, Brauer M, Nieuwenhuijsen MJ, Lupp G, Richardson EA, Astell-Burt T, Dimitrova D, Feng X, Sadeh M, Standl M, Heinrich J, Fuertes E (2017). Exploring pathways linking greenspace to health: theoretical and methodological guidance. Environ Res.

[24] Helliwell JF, Layard R, Sachs J (2018). World happiness report 2018.

[25] Kahneman D, Deaton A (2010). High income improves evaluation of life but not emotional well-being. Proc Natl Acad Sci.

[26] Miura T, Nagai S, Takeuchi M, Ichii K, Yoshioka H (2019). Improved characterisation of vegetation and land surface seasonal dynamics in central Japan with himawari-8 hypertemporal data. Sci Rep.

[27] IMF (2018) World Economic Outlook Database. https://www.imf.org/en/Publications/SPROLLS/world-economic-outlook-databases. Accessed 16 November 2020

[28] Easterlin RA, McVey LA, Switek M, Sawangfa O, Zweig JS (2010). The happiness-income paradox revisited. Proc Natl Acad Sci.

[29] Preacher KJ, Rucker DD, Hayes AF (2007). Addressing moderated mediation hypotheses: theory, methods, and prescriptions. Multivar Behav Res.

[30] Foley R, Kistemann T (2015). Blue space geographies: enabling health in place. Health Place.

[31] Raymond CM, Gottwald S, Kytta M (2016). Integrating multiple elements of environmental justice into urban blue space planning using public participation geographic information systems. Landsc Urban Plan.

[32] Groff E, McCord ES (2012). The role of neighborhood parks as crime generators. Secur J.

[33] Han B, Cohen DA, Derose KP, Li J, Williamson S (2018). Violent crime and park use in low-income urban neighborhoods. Am J Prev Med.

[34] Ugolini F, Massetti L, Calaza-Martínez P, Cariñanos P, Dobbs C, Ostoić SK, Marin AM, Pearlmutter D, Saaroni H, Šaulienė I, Simoneti M, Verlič A, Vuletić D, Sanesi G (2020). Effects of the Covid-19 pandemic on the use and perceptions of urban green space: an international exploratory study. Urban For Urban Green.

[35] Lolli S, Chen Y-C, Wang S-H, Vivone G (2020). Impact of meteorological conditions and air pollution on Covid-19 pandemic transmission in Italy. Sci Rep.

[36] Hedblom M, Gunnarsson B, Iravani B, Knez I, Schaefer M, Thorsson P, Lundström JN (2019). Reduction of physiological stress by urban green space in a multisensory virtual experiment. Sci Rep.

[37] Ewing RH (2008). Characteristics, causes, and effects of sprawl: a literature review. Urban ecology.

[38] Liu X, Huang Y, Xu X, Li X, Li X, Ciais P, Lin P, Gong K, Ziegler AD, Chen A, Gong P, Chen J, Hu G, Chen Y, Wang S, Wu Q, Huang K, Estes L, Zeng Z (2020). High-spatiotemporal-resolution mapping of global urban change from 1985 to 2015. Nat Sustain.

[39] Allen CD, Macalady AK, Chenchouni H, Bachelet D, McDowell N, Vennetier M, Kitzberger T, Rigling A, Breshears DD, Hogg EH, Gonzalez P, Fensham R, Zhang Z, Castro J, Demidova N, Lim J-H, Allard G, Running SW, Semerci A, Cobb N (2010). A global overview of drought and heat-induced tree mortality reveals emerging climate change risks for forests. For Ecol Manag.

[40] Pretzsch H, Biber P, Uhl E, Dahlhausen J, Schütze G, Perkins D, Rötzer T, Caldentey J, Koike T, van Con T, Chavanne A, du Toit B, Foster K, Lefer B (2017). Climate change accelerates growth of urban trees in metropolises worldwide. Sci Rep.

[41] Nowak DJ, Greenfield EJ, Hoehn RE, Lapoint E (2013). Carbon storage and sequestration by trees in urban and community areas of the United States. Environ Pollut.

[42] Houlden V, Weich S, Jarvis S (2017). A cross-sectional analysis of green space prevalence and mental wellbeing in England. BMC Public Health.

[43] Seiferling I, Naik N, Ratti C, Proulx R (2017). Green streets – quantifying and mapping urban trees with street-level imagery and computer vision. Landsc Urban Plan.

[44] Huete AR (1988). A soil-adjusted vegetation index (SAVI). Remote Sens Environ.

[45] Jiang Z, Huete AR, Didan K, Miura T (2008). Development of a two-band enhanced vegetation index without a blue band. Remote Sens Environ.

[46] Buchhorn M, Smets B, Bertels L, De Roo B, Lesiv M, Tsendbazar N-E, Herold M, Fritz S (2020) Copernicus Global Land Service: Land Cover 100m: Collection 3 Epoch 2015, Globe. https://lcviewer.vito.be/download

