# Supplementary material for: Urban green space and happiness in developed countries
Source: EPJ Data Sci. 2021 May 30;10(1):28. doi: 10.1140/epjds/s13688-021-00278-7 (PMC8164893; doi:10.1140/epjds/s13688-021-00278-7)
Supplement: Supplementary file 1 — Supporting figures and tables for the main manuscript (PDF 679 kB) [file 13688_2021_278_MOESM1_ESM.pdf]

# Supplementary Information

Oh-Hyun Kwon[1, *], Inho Hong[2, *], Jeasurk Yang[3], Donghee Yvette Wohn[4], Woo-Sung Jung[1, 5, 6, †], and Meeyoung Cha[7, 8, ‡]

[1]Department of Physics, Pohang University of Science and Technology, Pohang 37673, Republic of Korea.
[2]Center for Humans and Machines, Max Planck Institute for Human Development, Berlin 14195, Germany.
[3]Department of Geography, National University of Singapore, Singapore 119260, Singapore.
[4]Department of Informatics, New Jersey Institute of Technology, Newark, NJ 07103, USA.
[5]Department of Industrial and Management Engineering, Pohang University of Science and Technology, Pohang 37673, Republic of Korea.
[6]Asia Pacific Center for Theoretical Physics, Pohang 37673, Republic of Korea.
[7]Data Science Group, Institute for Basic Science, Daejeon 34126, Republic of Korea.
[8]School of Computing, Korea Advanced Institute of Science and Technology, Daejeon 34141, Republic of Korea.
[*]These authors contributed equally to this work: Oh-Hyun Kwon and Inho Hong
[†]wsjung@postech.ac.kr
[‡]mcha@ibs.re.kr

## Section S1. Data

### Section S1.1. Data description

Table S1 and S2 describe the dataset used in this study. The happiness scores were obtained from the World Happiness Report, which was averaged over three years to adjust for short-term fluctuations. The average happiness score is 6.373, with a maximum of 7.769 for Finland and a minimum of 4.549 for Iran. UGS is calculated from Sentinel-2 satellite imagery data, and GDP per capita (PPP) data is obtained from the IMF estimation.

We used the data of 60 developed countries selected by comparing the HDI of the countries. Andorra, Bahamas, Barbados, Brunei, Cyprus, Lichtenstein, Palau, and Seychelles are excluded from the analysis due to a lack of data for happiness.

| Country | City counts | Population [%] | Happiness | UGS | log-GDP |
|---|---|---|---|---|---|
| Finland | 1 | 28.23 | 7.77 | 5.73 | 10.70 |
| Iceland | 1 | 38.05 | 7.49 | 5.47 | 10.87 |
| Lithuania | 1 | 19.25 | 6.15 | 5.46 | 10.44 |
| New Zealand | 1 | 34.57 | 7.31 | 5.33 | 10.60 |
| Slovenia | 1 | 13.99 | 6.12 | 5.32 | 10.45 |
| Croatia | 1 | 19.82 | 5.43 | 5.23 | 10.11 |
| Montenegro | 1 | 31.07 | 5.52 | 5.21 | 9.85 |
| Italy | 1 | 7.21 | 6.22 | 5.17 | 10.56 |
| Slovakia | 2 | 12.25 | 6.20 | 5.16 | 10.46 |
| Estonia | 1 | 33.12 | 5.89 | 5.15 | 10.39 |
| United States | 3 | 12.76 | 6.89 | 5.13 | 11.03 |
| Latvia | 1 | 32.95 | 5.94 | 5.05 | 10.28 |
| Sweden | 2 | 15.01 | 7.34 | 5.00 | 10.88 |
| Switzerland | 4 | 10.87 | 7.48 | 4.98 | 11.04 |
| Norway | 1 | 12.80 | 7.54 | 4.97 | 11.19 |
| Canada | 1 | 18.26 | 7.28 | 4.96 | 10.80 |
| Serbia | 1 | 23.99 | 5.60 | 4.93 | 9.67 |
| Poland | 4 | 10.09 | 6.16 | 4.88 | 10.34 |
| Germany | 5 | 10.54 | 7.02 | 4.79 | 10.85 |
| Hungary | 1 | 17.89 | 5.82 | 4.78 | 10.31 |

**Table S1.** Data used in the study. Countries are ordered by UGS. We aggregate city-level data to cover at least 10% of total population.

| Country | City counts | Population [%] | Happiness | UGS | log-GDP |
|---|---|---|---|---|---|
| Czech Republic | 1 | 12.13 | 6.85 | 4.75 | 10.50 |
| Portugal | 3 | 11.67 | 5.69 | 4.72 | 10.32 |
| Bulgaria | 1 | 18.69 | 5.01 | 4.70 | 10.02 |
| Australia | 1 | 19.55 | 7.23 | 4.69 | 10.86 |
| Netherlands | 3 | 10.71 | 7.49 | 4.52 | 10.91 |
| Luxembourg | 1 | 30.40 | 7.09 | 4.49 | 11.59 |
| Ireland | 1 | 11.62 | 7.02 | 4.36 | 11.24 |
| United Kingdom | 1 | 13.42 | 7.05 | 4.28 | 10.72 |
| Trinidad and Tobago | 1 | 12.76 | 6.19 | 4.25 | 10.46 |
| Uruguay | 1 | 39.42 | 6.29 | 4.16 | 10.06 |
| Spain | 1 | 13.97 | 6.35 | 4.15 | 10.59 |
| Russia | 2 | 12.50 | 5.65 | 4.12 | 10.24 |
| Belarus | 1 | 20.82 | 5.32 | 4.12 | 9.82 |
| Austria | 1 | 21.42 | 7.25 | 4.11 | 10.83 |
| Panama | 1 | 27.74 | 6.32 | 4.06 | 10.15 |
| Kazakhstan | 1 | 11.13 | 5.81 | 4.06 | 10.19 |
| Albania | 1 | 23.32 | 4.72 | 4.06 | 9.51 |
| Mauritius | 1 | 29.03 | 5.89 | 3.94 | 10.05 |
| Costa Rica | 1 | 32.68 | 7.17 | 3.93 | 9.79 |
| Belgium | 1 | 10.58 | 6.92 | 3.91 | 10.76 |
| Denmark | 1 | 10.78 | 7.60 | 3.89 | 10.82 |
| Romania | 2 | 10.93 | 6.07 | 3.87 | 10.13 |
| France | 1 | 10.62 | 6.59 | 3.72 | 10.72 |
| Malaysia | 1 | 12.19 | 5.34 | 3.64 | 10.31 |
| Argentina | 2 | 10.13 | 6.09 | 3.33 | 9.98 |
| Turkey | 1 | 18.34 | 5.37 | 3.28 | 10.05 |
| Greece | 1 | 24.11 | 5.29 | 3.28 | 10.30 |
| Malta | 3 | 13.05 | 6.73 | 3.17 | 10.64 |
| Chile | 1 | 30.54 | 6.45 | 3.05 | 10.15 |
| Japan | 1 | 10.63 | 5.89 | 3.03 | 10.63 |
| Iran | 1 | 10.86 | 4.55 | 2.90 | 9.91 |
| Singapore | 1 | 100.00 | 6.26 | 2.87 | 11.45 |
| South Korea | 1 | 19.00 | 5.89 | 2.70 | 10.64 |
| Israel | 1 | 10.75 | 7.14 | 2.65 | 10.53 |
| United Arab Emirates | 1 | 35.82 | 6.82 | 2.23 | 11.17 |
| Saudi Arabia | 1 | 19.49 | 6.37 | 2.06 | 10.95 |
| Oman | 1 | 32.66 | 6.85 | 2.05 | 10.73 |
| Kuwait | 1 | 12.79 | 6.02 | 1.91 | 11.22 |
| Qatar | 1 | 39.77 | 6.37 | 1.23 | 11.82 |
| Bahrain | 1 | 38.47 | 6.20 | 0.54 | 10.86 |

**Table S2.** Data used in the study. Countries are ordered by UGS. We aggregate city-level data to cover at least 10% of total population.

## Section S1.2. Normality of the data

We test normality for logarithmic NDVI per capita (UGS) and logarithmic GDP per capita (GDP) after taking a logarithm. The tests show that UGS has a unimodal distribution, but it does not follow a normal distribution, whereas GDP follows a normal distribution. The p-values of the D'Agostino and Pearson's test for UGS and GDP are 0.005 and 0.664, respectively. Also, the p-values of the Shapiro-Wilk test for UGS and GDP are 0.001 and 0.952, respectively. The linear Q-Q plot (Fig. S1) also confirms the normality of the GDP distribution.

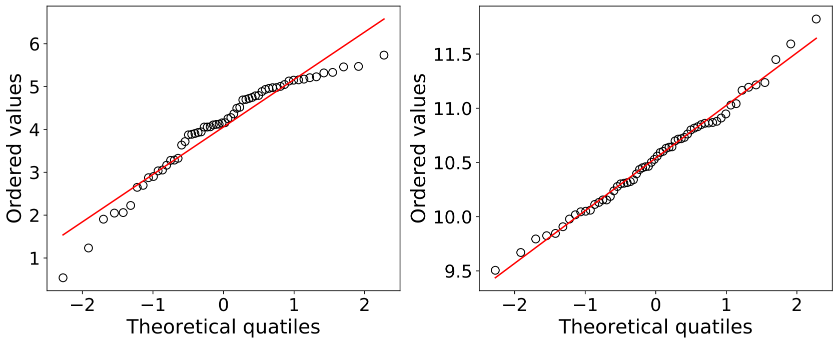


**Fig. S1.** Q-Q plots of logarithmic NDVI per capita and logarithmic GDP per capita.

# Section S2. Robustness of urban green space measures

The result of the regression is robust for any green space measure. In table S3, all nine urban green space measures explain happiness along with GDP, while logarithmic NDVI per capita (UGS) in the model (5) displays the highest value of adjusted $R^2$ compared to other models.

| | (1) | (2) | (3) | (4) | (5) | (6) | (7) | (8) | (9) |
|---|---|---|---|---|---|---|---|---|---|
| GDPLN | 1.0684$^{***}$ | 1.0198$^{***}$ | 1.1118$^{***}$ | 1.0333$^{***}$ | 1.1319$^{***}$ | 1.1138$^{***}$ | 1.0347$^{***}$ | 1.1001$^{***}$ | 1.0292$^{***}$ |
| | (0.6455) | (0.6291) | (0.6507) | (0.6264) | (0.6234) | (0.6501) | (0.6266) | (0.6453) | (0.6256) |
| GreenRatio | 0.0101$^{**}$ | - | - | - | - | - | - | - | - |
| | (0.0177) | | | | | | | | |
| GreenperCapita | - | 0.0020$^{**}$ | - | - | - | - | - | - | - |
| | | (0.0031) | | | | | | | |
| NDVIMean | - | - | 1.8166$^{**}$ | - | - | - | - | - | - |
| | | | (2.8613) | | | | | | |
| NDVIperCapita | - | - | - | 0.0029$^{***}$ | - | - | - | - | - |
| | | | | (0.0042) | | | | | |
| NDVILN | - | - | - | - | 0.2249$^{***}$ | - | - | - | - |
| | | | | | (0.2643) | | | | |
| SAVIMean | - | - | - | - | - | 1.2267$^{**}$ | - | - | - |
| | | | | | | (1.9051) | | | |
| SAVIperCapita | - | - | - | - | - | - | 0.0019$^{***}$ | - | - |
| | | | | | | | (0.0028) | | |
| EVI2Mean | - | - | - | - | - | - | - | 0.9462$^{**}$ | - |
| | | | | | | | | (1.4709) | |
| EVI2perCapita | - | - | - | - | - | - | - | - | 0.0016$^{***}$ |
| | | | | | | | | | (0.0022) |
| Const | -5.1875*** | -4.5886*** | -5.8446*** | -4.7804*** | -6.4709*** | -5.8728*** | -4.7950*** | -5.6947*** | -4.7325*** |
| | (6.9109) | (6.6518) | (7.0963) | (6.6389) | (6.8998) | (7.0905) | (6.6423) | (7.0009) | (6.6259) |
| Adjusted $R^2$ | 0.4248 | 0.4407 | 0.4363 | 0.4466 | 0.4786 | 0.4379 | 0.4465 | 0.4378 | 0.4476 |
| Observations | 60 | 60 | 60 | 60 | 60 | 60 | 60 | 60 | 60 |

**Table S3.** Regression analysis of happiness with different green space measures, $^{***}p< 0.01$; $^{**}p< 0.05$; $^{*}p< 0.1$.

We check the robustness of UGS in explaining happiness with extra environmental variables. We retrieve the data for the ratio of forest land cover and protected areas from the dataset of UNSD. The ratio of forest land cover and protected areas show the Pearson correlations of 0.4929 and 0.4362 with UGS (p-value of 0.0001 and 0.0006), respectively. Although the ratio of forest land cover and protected area correlated with UGS, they fail to capture the relationship between green space and happiness as UGS do. The regression models (5-8) in Table S4 show that the ratio of forest land cover and protected areas does not improve the model for happiness even with the GDP variable, confirming that the models using UGS are robust for extra environmental variables.

| | (1) | (2) | (3) | (4) | (5) | (6) | (7) | (8) |
|---|---|---|---|---|---|---|---|---|
| GDP | 1.0208$^{***}$ | - | - | - | 1.1259$^{***}$ | 1.0475$^{***}$ | 1.0120$^{***}$ | 1.1594$^{***}$ |
| | (0.6758) | | | | (0.6341) | (0.6952) | (0.6871) | (0.6601) |
| UGS | - | 0.1338 | - | - | 0.2285$^{***}$ | - | - | 0.3032$^{***}$ |
| | | (0.3634) | | | (0.2711) | | | (0.3338) |
| Forest land cover | - | - | -0.0025 | - | - | 0.0028 | - | -0.0033 |
| | | | (0.0200) | | | (0.0161) | | (0.0165) |
| Protected area | - | - | - | 0.0033 | - | - | 0.0011 | -0.0038 |
| | | | | (1.0141) | | | (0.0112) | (0.0116) |
| Const | -4.3842$^{**}$ | -5.8183$^{***}$ | -6.4433$^{***}$ | -6.1857$^{***}$ | -6.4253$^{***}$ | -4.7509$^{**}$ | -4.3514$^{**}$ | -6.7746$^{***}$ |
| | (7.1240) | (1.5387) | (0.7365) | (0.8705) | (6.9869) | (7.4628) | (7.1878) | (7.2294) |
| Adjusted $R^2$ | 0.3803 | 0.0199 | -0.0130 | -0.0022 | 0.4759 | 0.3747 | 0.3710 | 0.4799 |
| Observations | 59 | 59 | 59 | 59 | 59 | 59 | 59 | 59 |

**Table S4.** Regression analysis of happiness with ratio of forest land cover and protected area variables. UAE is excluded from the analysis due to missing data. $^{***}p<0.01$; $^{**}p<0.05$; $^{*}p<0.1$.

## Section S3. Regional influence

As regional characteristics may affect the level of green space, we test the robustness of the green space-happiness relation for regional characteristics. Figure S2 describes the change of USG by latitude. Countries with a tropical climate such as Southeastern Asia, the Caribbean, and Eastern Africa show a relatively high UGS score. In contrast, Western Asian countries show a relatively low UGS score since they are in a dry climate. The UGS score further increases in higher latitudes.

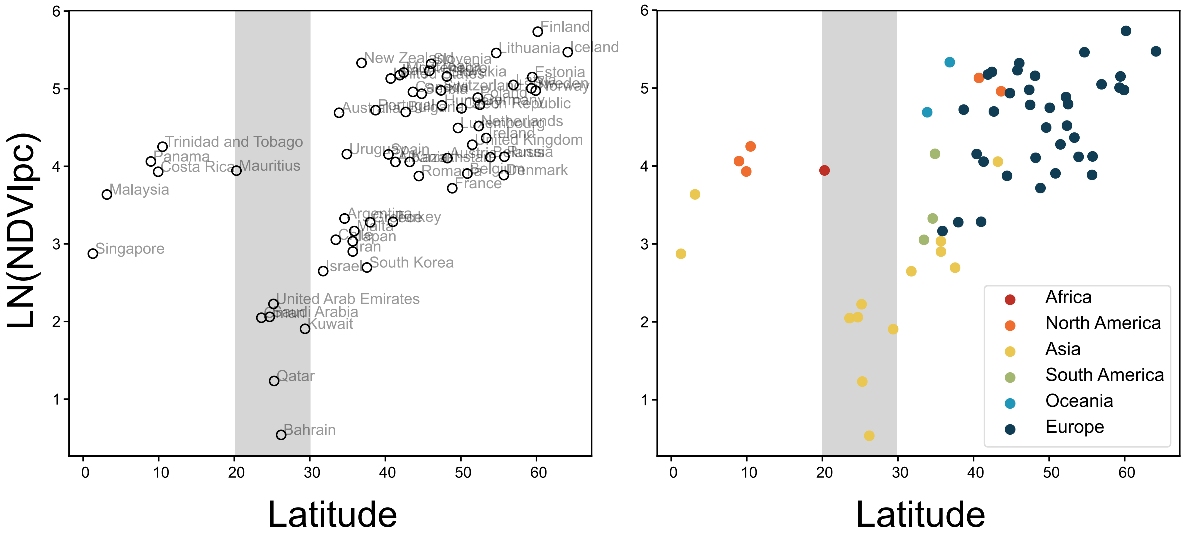


**Fig. S2.** Scatter plot of UGS and latitude with country (left) and continent (right) marked. Gray area represent the dry climate region.

In Table S5, model (3) includes the latitude of the most populated city, model (4-5) includes dummy variables that tell whether the countries in Western Asia or the dry climate region. These models show that including regional factors does not improve the model.

| | (1) | (2) | (3) | (4) | (5) |
|---|---|---|---|---|---|
| GDP | 1.0120*** | 1.1319*** | 1.1275*** | 1.1142*** | 1.1347*** |
| | (0.6603) | (0.6234) | (0.6413) | (0.6433) | (0.6297) |
| UGS | - | 0.2249*** | 0.2181** | 0.2585*** | 0.2055** |
| | | (0.2643) | (0.3302) | (0.3770) | (0.3641) |
| Latitude | - | - | 0.0009 | - | - |
| | | | (0.0250) | | |
| Western Asia | - | - | - | 0.1595 | - |
| | | | | (1.2679) | |
| Dry Climate | - | - | - | - | -0.0885 |
| | | | | | (1.1301) |
| Const | -4.2945** | -6.4709*** | -6.4326*** | -6.4422*** | -6.4081*** |
| | (6.9672) | (6.8998) | (7.0474) | (6.9518) | (7.0037) |
| Adjusted $R^2$ | 0.3832 | 0.4786 | 0.4695 | 0.4717 | 0.4702 |
| Observations | 60 | 60 | 60 | 60 | 60 |

**Table S5.** Regression analysis of happiness with region variables. ***p< 0.01; **p< 0.05; *p< 0.1.

## Section S4. Distribution of green space

Figure S3 describes the distribution of three green space measures. NDVIavg (average NDVI) is calculated by taking the mean NDVI values over the built-up area, representing how much greenery cities have. NDVIpc (average NDVI per capita) is obtained by dividing the total NDVI by the total population. NDVI per capita describes how much green space is provided to a population. However, NDVI per capita shows a skewed distribution, which is not appropriate for regression analysis. Therefore, we take a logarithm of NDVI per capita to get a unimodal distribution of the green space measure.

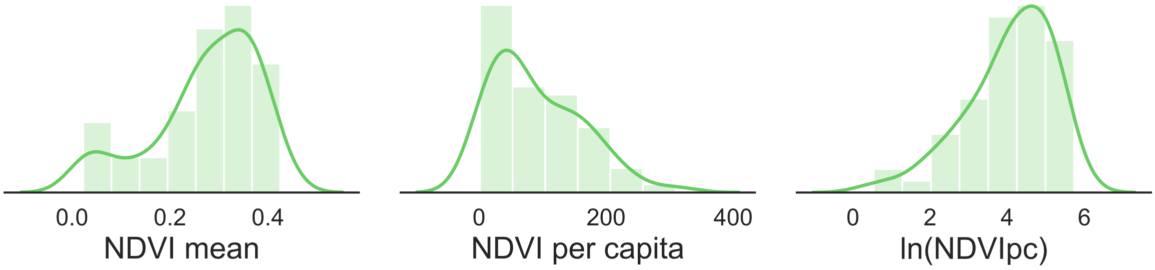


**Fig. S3.** Distribution plot of NDVI mean, NDVI per capita, logarithmic NDVI per capita

## Section S5. Residual analysis

We perform a residual analysis of the regression model in Table 1 to check whether the model is reasonable. First, we need to check the autocorrelation of the residuals by using Durbin-Watson statistics. The Durbin-Watson statistics show a value of 1.918, which indicates there are no autocorrelations between the residuals. Second, we check for the normality of the residuals. The distribution and Q-Q plot of the residuals shows that the residuals satisfy the normality condition. Finally, we check for the equality of variance by finding outliers using Cook's distance. The figure shows that every point has a value of less than 1, indicating acceptable values.

## Section S6. The effect of GDP on the green-happiness relation

We can check for a similar result of Fig. 3(c) in the manuscript by calculating the Pearson correlation instead of the regression coefficient. Figure S5 shows a similar diminishing effect of green space as the group contains lower GDP countries. In contrast, log-GDP shows the most strong correlations for the entire dataset containing lower GDP groups.

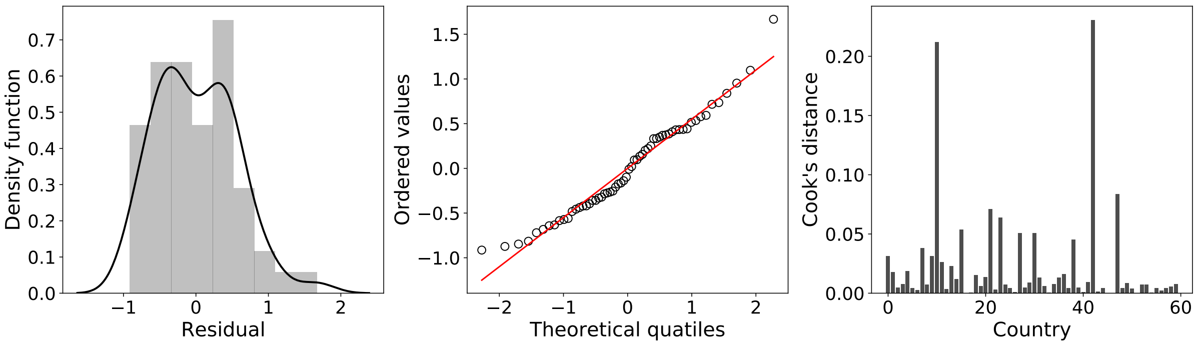


**Fig. S4.** Residual analysis of the regression model. (left) The distribution, (middle) Q-Q plot, and (right) cook's distance of residuals.

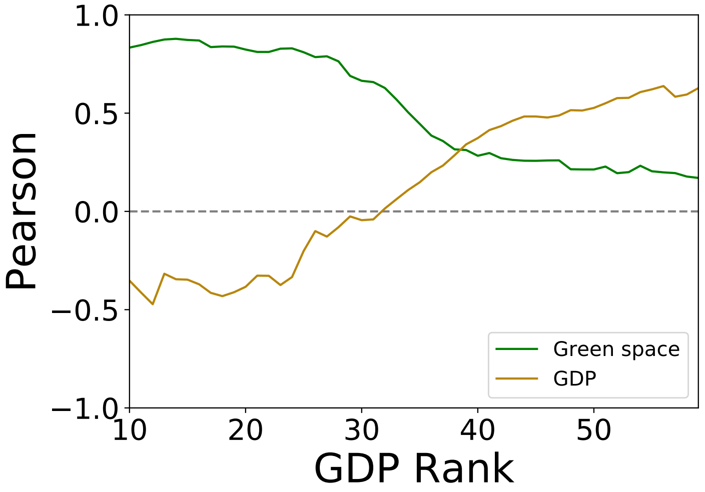


**Fig. S5.** Changes of the Pearson correlation between urban green space and happiness for different sets of GDP rank with increasing window size from top 10 to 60.

## Section S7. Happiness Report variables

To describe the happiness scores, World Happiness Report provides six main variables: *GDP, social support, life expectancy, freedom, generosity*, and *corruption perceptions*. *Social support* and *freedom* are based on binary responses (yes or no) to World Gallup Poll (WGP) questions; "If you were in trouble, do you have relatives or friends you can count on to help you whenever you need them, or not?", and "Are you satisfied or dissatisfied with your freedom to choose what you do with your life?", respectively. *generosity* is the residual of regression for responses for a WGP question "Have you donated money to a charity in the past month?" on GDP per capita. *Corruption perceptions* is based on the response to WGP question, "Is corruption widespread throughout the government or not?" and "Is corruption widespread within businesses or not?" *Life expectancy* is based on the Global Health Observatory data from World Health Organization (WHO).

Here, we check how our analyses fit into these six variables. The data of 6 variables are retrieved from the World Happiness Report, and we took a 3-year average. Figure S6 shows the scatter plots between UGS and six variables in the World Happiness Report. Note that the scatter plot between UGS and social support presents a relatively strong Pearson correlation of 0.4329, while the other variables are not correlated with UGS. Therefore, we speculate that UGS is connected with the social support variable, which should be considered in constructing regression models.

Since the data for corruption perceptions is missing for six countries, and it seems to fail to explain happiness well for developed countries' dataset, we checked the regression with and without corruption perception. The regression model (1) shows that UGS can explain happiness in place of social support, even although the adjusted R-square value is smaller compared to model (2), which includes social

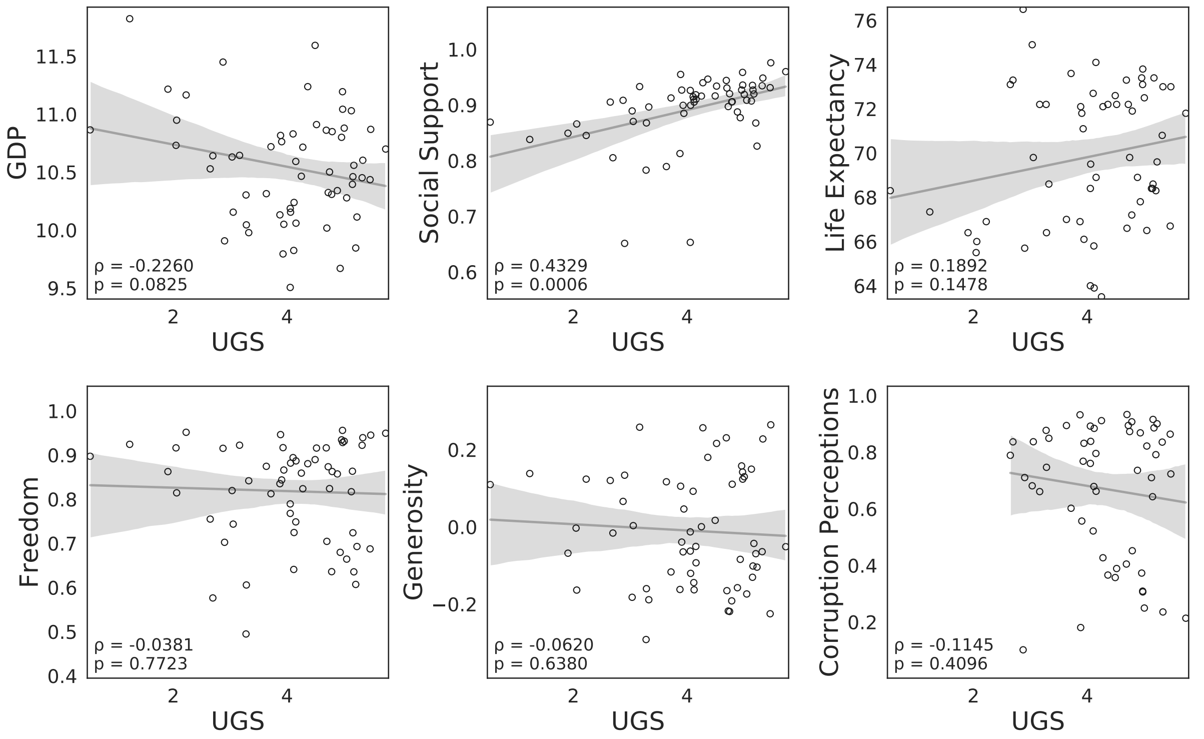


**Fig. S6.** Scatter plot between UGS and variables in World Happiness Report. $\rho$ indicates the Pearson correlation.

support. Furthermore, model (3), which includes both UGS and social support, shows that UGS loses its explainability while social support. The same result can be found in the model (4-6).

# Section S8. Regressions with extra socioeconomic variables

While we use variables from the World Happiness Report, many other socioeconomic variables may be related to happiness. Here, we examine the robustness of the relationship between urban green space and happiness for extra control variables: health expenditure, unemployment, education and gender inequality. The data for health expenditure (percentage of current health expenditure compared to GDP) and the unemployment rate are retrieved from the dataset of UNSD. Education Index and Gender Inequality Index are retrieved from the Human Development Report 2016 and the UNDP Human Development Reports 2020.

Fig. S7 displays the relationship between adopted extra socioeconomic variables, happiness, social support, and UGS. The health expenditure and the education index show positive correlations with happiness and social support, while the unemployment rate and the gender inequality index show negative correlations with happiness and social support. Note that health expenditure and education index show significantly high Pearson correlations with UGS.

We investigate whether adopting extra socioeconomic variables would affect our conclusions. Table S7 show the regression models with extra control variables. When every control variables are included (i.e., the models (3-4)), UGS does not have significance in explaining happiness since UGS has strong multicollinearity with the health expenditure (i.e., the Pearson correlation $\rho$ is 0.47) and the education index (i.e., $\rho = 0.57$) as shown in Fig. S7. When this multicollinearity is removed by excluding the health expenditure and the education index, adopting UGS increases the adjusted R-square value, and UGS has significance in the model (6). Therefore, the green space-happiness relation is robust for including extra control variables.

In Table S8, we check the robustness of the regression models for social support. UGS significantly explains social support along with every other variable in the models (1-5). Furthermore, in the models (6-7) with every variable, adopting UGS significantly increases the adjusted R-square value. In conclusion, the explanatory power of UGS for social support is robust for including extra control variables, and we

| | Without corruption perceptions | | | With corruption perceptions | | |
|---|---|---|---|---|---|---|
| | (1) | (2) | (3) | (4) | (5) | (6) |
| GDP | 0.5187$^{***}$ | 0.2388$^{*}$ | 0.2779$^{*}$ | 0.4694$^{**}$ | 0.1482 | 0.1512 |
| | (0.6508) | (0.5344) | (0.5991) | (0.9038) | (0.8178) | (0.8243) |
| UGS | 0.1690$^{***}$ | - | 0.0339 | 0.1729$^{**}$ | - | 0.0442 |
| | (0.2263) | | (0.2290) | (0.3255) | | (0.3018) |
| Social Support | - | 5.1863$^{***}$ | 4.8452$^{***}$ | - | 5.2457$^{***}$ | 4.9787$^{***}$ |
| | | (3.6667) | (4.3514) | | (4.2056) | (4.6136) |
| Life Expectancy | 0.0606$^{***}$ | 0.0556$^{***}$ | 0.0535$^{***}$ | 0.0558$^{**}$ | 0.0557$^{**}$ | 0.0580$^{**}$ |
| | (0.0872) | (0.0733) | (0.0751) | (0.1115) | (0.0929) | (0.0950) |
| Freedom | 2.4609$^{***}$ | 1.7472$^{***}$ | 1.7652$^{***}$ | 2.2086$^{***}$ | 1.5238$^{**}$ | 1.5036$^{**}$ |
| | (2.6342) | (2.3277) | (2.3463) | (2.9002) | (2.5320) | 2.5549) |
| Generosity | 0.6584 | 1.0520$^{**}$ | 1.0346$^{**}$ | 0.60031 | 1.0493$^{**}$ | 1.0563$^{**}$ |
| | (2.0066) | (1.7401) | (1.7555) | (2.2309) | (1.9306) | (1.4819) |
| Corruption Perceptions | - | - | - | -0.3589 | -0.3109 | -0.3039 |
| | | | | (1.7389) | (8.6572) | (8.7725) |
| Const | -6.0323$^{***}$ | -6.0873$^{***}$ | -6.2005$^{***}$ | -4.7584$^{*}$ | -4.8097$^{**}$ | -4.9458$^{**}$ |
| | (7.1740) | (6.0731) | (6.1610) | (10.2984) | (8.5672) | (8.7725) |
| Adjusted $R^2$ | 0.6753 | 0.7638 | 0.7609 | 0.6823 | 0.7730 | 0.7698 |
| Observations | 59 | 59 | 59 | 54 | 54 | 54 |

**Table S6.** Regression analysis of happiness with (1-3) 5 variables and (4-6) 6 variables in the World Happiness Report. We separated the models with corruption perceptions since few countries are missing data: Oman is excluded from the model (1-3), and Bahrain, Kuwait, Oman, Qatar, Saudi Arabia, and the United Arab Emirates are excluded from the model (4-6). $^{***}$p< 0.01; $^{**}$p< 0.05; $^{*}$p< 0.1.

construct the moderated mediation model for happiness, UGS, GDP and social support based on this robustness.

# Section S9. Moderated mediation model for regression

A moderation and mediation technique can provide a more sophisticated regression model that describes more detailed pathways behind simple regression.

The mediation model describes indirect effects of mediation variables by a two-staged regression model. We apply the moderation model for log-GDP since we checked that the regression analyses for social support depend on the GDP value, which can be described with a cross-term. We can set up the regression model as follows:

$$H = \beta_0 + \beta_1 M + \beta_2 S + \beta_3 SM$$
$$S = \beta_4 + \beta_5 G$$

Now, the mediation model can be validated by comparing the multilinear regression model with its explanation of power. We check whether green space is related to happiness through social support.

In Table S9, the models (1-3) describe the effect of UGS and social support on happiness. UGS and social support can explain happiness and GDP in the model (1) and (2). However, UGS loses its explainability when we include both UGS and social support in the model (3), which implies that UGS only indirectly affects happiness compared to social support. Note that our mediation model was valid for GDP, so the moderated mediation model would be more appropriate.

The moderation effect of the model can be validated by calculating the regression model with a cross-term. We check several moderation models for different pairwise relations: moderation for green-social, social-happiness, and green-happiness. We find that the moderation effect emerges for the social-happiness relation with a higher adjusted R-square value and a significantly low p-value (Table S9 model (4)). Therefore, we conclude that green space is related to happiness through social support, and GDP moderates social support on happiness.

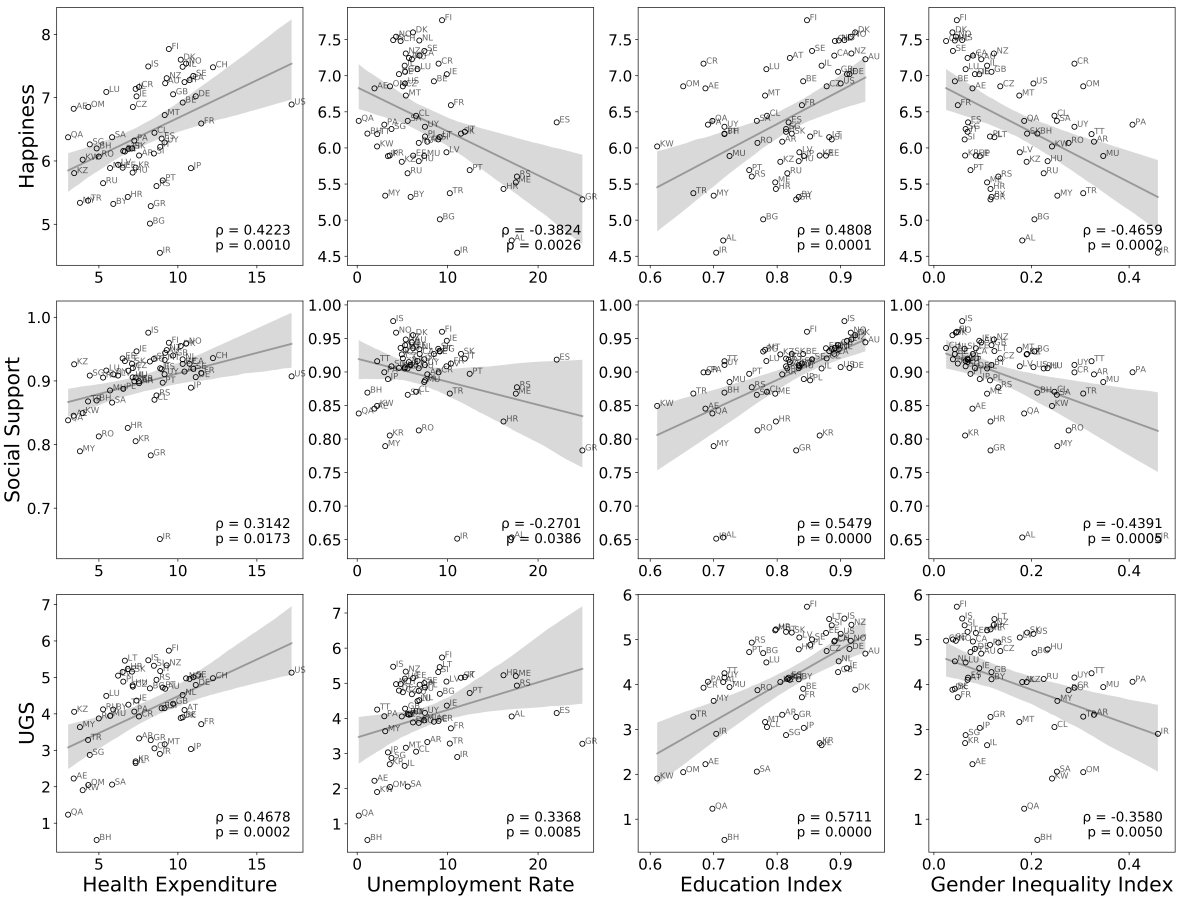


**Fig. S7.** Scatter plot of extra variables with happiness (top), social support (middle) and UGS (bottom). $\rho$ indicates the Pearson correlations and p indicates p-values.

# Section S10. Derivation of happiness equation

How much do we need green space to increase our happiness? Since our analyses are based on regression models, we can provide a numerical estimation of the required green space corresponding to a certain increment of happiness. Let us consider our final regression model:

$$H = \beta_0 + \beta_1 \ln M + \beta_2 S + \beta_3 S \ln M,$$
$$S = \beta_4 + \beta_5 \ln G,$$

where $H$ is the happiness score, $M$ is GDP per capita, $S$ is social support, and $G$ is UGS. If we substitute social support into the equation, we obtain the following equation as

$$H = \beta_0' + (\beta_1' + \beta_2' \ln M) \ln G + \beta_3' \ln M.$$

If we assume that the value of GDP per capita stays the same, we can solve a fraction of green space change to increase a certain amount of happiness. We set the happiness score change to 0.0546, which is an average value for upgrading one rank.

$$\frac{G_f}{G_i} = \exp\left(\frac{\Delta H}{\beta_1' + \beta_2' \ln M}\right).$$

| Country | Green Space [%] | NDVI per capita | GDP per capita [dollar] |
|---|---|---|---|
| Qatar | 14.50 | 0.4981 | 7556 |
| Luxembourg | 16.00 | 14.3032 | 6004 |
| Singapore | 17.10 | 3.0292 | 5199 |
| Ireland | 19.04 | 14.9560 | 4205 |

| | | | |
|---|---|---|---|
| Kuwait | 19.26 | 1.2950 | 4115 |
| Norway | 19.49 | 28.2059 | 4026 |
| United Arab Emirates | 19.79 | 1.8334 | 3914 |
| Switzerland | 21.24 | 30.8228 | 3461 |
| United States | 21.41 | 36.1908 | 3416 |
| Saudi Arabia | 22.50 | 1.7651 | 3149 |
| Netherlands | 23.05 | 21.1280 | 3032 |
| Sweden | 23.52 | 35.0743 | 2941 |
| Iceland | 23.68 | 56.2871 | 2909 |
| Bahrain | 23.75 | 0.4071 | 2896 |
| Australia | 23.80 | 25.8874 | 2888 |
| Germany | 23.99 | 28.9519 | 2853 |
| Austria | 24.31 | 14.7517 | 2797 |
| Denmark | 24.53 | 11.9521 | 2761 |
| Canada | 24.81 | 35.3155 | 2716 |
| Belgium | 25.48 | 12.6587 | 2615 |
| Oman | 26.06 | 2.0204 | 2535 |
| France | 26.29 | 10.8164 | 2504 |
| United Kingdom | 26.37 | 18.9955 | 2495 |
| Finland | 26.68 | 82.4975 | 2455 |
| Malta | 27.81 | 6.5911 | 2325 |
| South Korea | 27.90 | 4.1332 | 2315 |
| Japan | 28.12 | 5.8425 | 2292 |
| New Zealand | 28.75 | 59.4447 | 2229 |
| Spain | 29.00 | 18.4593 | 2205 |
| Italy | 29.80 | 52.6365 | 2133 |
| Israel | 30.58 | 4.3248 | 2069 |
| Czech Republic | 31.29 | 36.0511 | 2015 |
| Trinidad and Tobago | 32.34 | 22.7089 | 1941 |
| Slovakia | 32.44 | 56.3721 | 1934 |
| Slovenia | 32.72 | 66.8141 | 1916 |
| Lithuania | 33.22 | 78.0579 | 1885 |
| Estonia | 34.51 | 59.4736 | 1810 |
| Poland | 36.40 | 48.1184 | 1715 |
| Portugal | 37.03 | 41.6695 | 1686 |
| Malaysia | 37.40 | 14.1884 | 1670 |
| Hungary | 37.63 | 45.0125 | 1660 |
| Greece | 37.85 | 10.0445 | 1652 |
| Latvia | 38.86 | 60.5196 | 1611 |
| Russia | 40.60 | 25.0656 | 1549 |
| Kazakhstan | 43.12 | 24.8977 | 1470 |
| Panama | 44.84 | 26.0437 | 1424 |
| Chile | 44.84 | 9.5004 | 1424 |
| Romania | 46.17 | 22.2308 | 1391 |
| Croatia | 47.31 | 88.2509 | 1365 |
| Uruguay | 50.84 | 32.4731 | 1295 |
| Mauritius | 51.53 | 26.5892 | 1283 |
| Turkey | 51.91 | 13.8637 | 1276 |
| Bulgaria | 54.05 | 59.3985 | 1241 |
| Argentina | 57.43 | 15.9950 | 1193 |
| Iran | 64.68 | 11.7746 | 1112 |
| Montenegro | 72.63 | 132.6388 | 1045 |
| Belarus | 75.81 | 46.5536 | 1023 |
| Costa Rica | 80.85 | 41.1266 | 993 |
| Serbia | 111.22 | 154.5325 | 876 |
| Albania | 213.00 | 122.9685 | 744 |

**Table S10.** Required green spaces and GDP to increase happiness by 0.0546.

| | (1) | (2) | (3) | (4) | (5) | (6) |
|---|---|---|---|---|---|---|
| UGS | - | 0.2247*** | - | 0.1174 | - | 0.2054** |
| | | (0.2680) | | (0.3432) | | (0.3103) |
| GDP | 0.9483*** | 1.0686*** | 0.5780*** | 0.6812*** | 0.5041** | 0.7434*** |
| | (0.7092) | (0.6677) | (0.8492) | (0.8948) | (0.9105) | (0.9364) |
| Unemployment | - | - | -0.0415** | -0.0443** | -0.0370* | -0.0403** |
| | | | (0.0746) | (0.0745) | (0.0801) | (0.0762) |
| Gender Inequality Index | - | - | -1.1271 | -0.9583 | -2.6266*** | -1.3500 |
| | | | (4.5005) | (4.4919) | (3.6477) | (3.9617) |
| Health Expenditure | - | - | 0.0919*** | 0.0844** | - | - |
| | | | (0.1326) | (0.1333) | | |
| Education Index | - | - | 1.0169 | 0.2253 | - | - |
| | | | (5.6305) | (6.0460) | | |
| Const | -3.6078* | -5.7865*** | -0.7550 | -1.6253 | -1.7681 | -1.7666 |
| | (7.5034) | (7.3723) | (11.5649) | (11.7516) | (10.3076) | (11.1418) |
| Adjusted $R^2$ | 0.3270 | 0.4315 | 0.5081 | 0.5163 | 0.4008 | 0.4612 |
| Observations | 58 | 58 | 58 | 58 | 58 | 58 |

**Table S7.** Regression analysis for happiness with extra control variables. Albania and Montenegro are excluded from the analysis due to missing data. ***p< 0.01; **p< 0.05; *p< 0.1.

| | (1) | (2) | (3) | (4) | (5) | (6) | (7) |
|---|---|---|---|---|---|---|---|
| UGS | 0.0288*** | 0.0228*** | 0.0303*** | 0.0149** | 0.0193*** | - | 0.0234*** |
| | (0.0209) | (0.0250) | (0.0219) | (0.0253) | (0.0217) | | (0.0266) |
| GDP | 0.432*** | - | - | - | - | -0.0148 | 0.0055 |
| | (0.0506) | | | | | (0.0726) | (0.0696) |
| Health Expenditure | - | 0.0021 | - | - | - | 0.0016 | 0.0001 |
| | | (0.0107) | | | | (0.0113) | (0.0103) |
| Unemployment | - | - | -0.0041*** | - | - | -0.0032** | -0.0038** |
| | | | (0.0053) | | | (0.0063) | (0.0058) |
| Education Index | - | - | - | 0.2576*** | - | 0.2138* | 0.0585 |
| | | | | (0.3609) | | (0.4830) | (0.4714) |
| Gender Inequality Index | - | - | - | - | -0.1907*** | -0.1844* | 0.1517* |
| | | | | | (0.2334) | (0.3836) | (0.3489) |
| Const | 0.3247** | 0.7883*** | 0.8048*** | 0.6276*** | 0.8487*** | 0.9202*** | 0.7479*** |
| | (0.5597) | (0.1020) | (0.0884) | (0.2520) | (0.1082) | (0.9840) | (0.9113) |
| Adjusted $R^2$ | 0.3765 | 0.2507 | 0.3550 | 0.3416 | 0.3675 | 0.3217 | 0.4458 |
| Observations | 57 | 57 | 57 | 57 | 57 | 57 | 57 |

**Table S8.** Regression analysis for social support with extra variables. Albania, Montenegro and Oman are excluded from the analysis due to missing data. ***p< 0.01; **p< 0.05; *p< 0.1.

| | (1) | (2) | (3) | (4) |
|---|---|---|---|---|
| log-GDP | 1.1321*** | 0.7168*** | 0.7936*** | -4.1830** |
| | (0.6193) | (0.5687) | (0.6336) | (6.566) |
| UGS | 0.2457*** | - | 0.0782 | - |
| | (0.2697) | | (0.2863) | |
| Social Support | - | 6.3899*** | 5.5731*** | -50.1512** |
| | | (4.4264) | (5.3363) | (75.625) |
| log-GDP:Social Support | - | - | - | 5.5583*** |
| | | | | (7.423) |
| Const | -6.5695*** | -6.8962*** | -7.2953*** | 42.8656** |
| | (6.8599) | (5.9001) | (6.0703) | (66.687) |
| Adjusted $R^2$ | 0.4912 | 0.6057 | 0.6071 | 0.6550 |
| Observations | 59 | 59 | 59 | 59 |

**Table S9.** Regression analysis for the moderated mediation model. Coefficient of GDP-Social Support represent cross term of GDP and social support. ***p< 0.01; **p< 0.05; *p< 0.1.

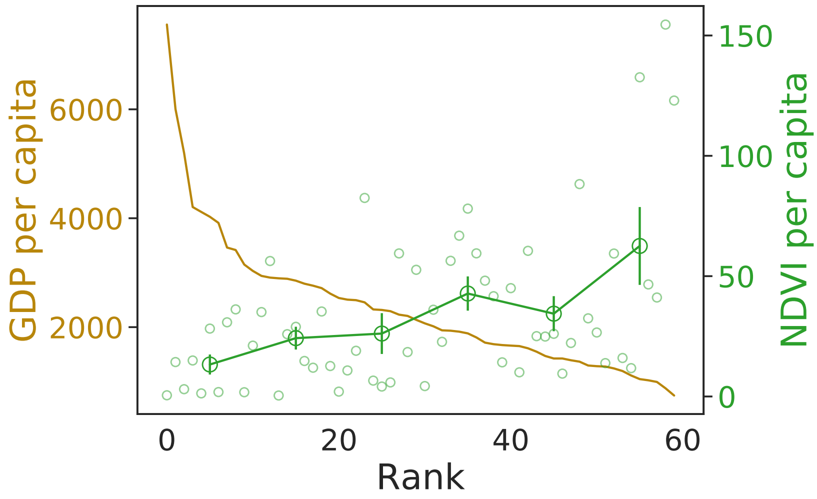


**Fig. S8.** Required GDP per capita (yellow) and NDVI per capita (green) to increase average amount of happiness to rank up by 1.
